# Mid‐Term Tibial Tunnel Enlargement and Graft Maturation After ACL Reconstruction: A 5‐Year Follow‐Up Linking Morphological Dynamics to Knee Function

**DOI:** 10.1111/os.70287

**Published:** 2026-03-19

**Authors:** Wenbo Tang, Feng Gao, Tao Li, Xiaohan Zhang, Bingying Zhang, Jingyi Sun, Feng Qu, Mingze Liu, Jingbin Zhou

**Affiliations:** ^1^ Department of Sports Medicine and Rehabilitation Beijing Chaoyang Hospital, Capital Medical University Beijing China; ^2^ School of Sports Medicine and Rehabilitation Beijing Sport University Beijing China; ^3^ National Institute of Sports Medicine Beijing China; ^4^ National Institute of Key Laboratory of Sports Trauma and Rehabilitation of General Administration of Sport of China Beijing China

**Keywords:** anterior cruciate ligament reconstruction, graft maturation, knee function, tibial tunnel enlargement

## Abstract

**Objective:**

Anterior cruciate ligament reconstruction (ACLR) with autologous hamstring tendon is the standard treatment for ACL rupture. However, tibial tunnel enlargement and delayed graft maturation may affect long‐term outcomes. Evidence on their mid‐ to long‐term associations with clinical recovery remains limited. The main objectives of this study include: (i) analyze longitudinal changes in tibial tunnel morphology after single‐bundle ACLR; (ii) quantitatively evaluate graft maturation at different tunnel regions using the signal intensity ratio (SIR) from MRI; and (iii) examine the correlations between tibial tunnel enlargement, graft healing, and clinical outcomes.

**Methods:**

A retrospective study was conducted on 35 patients who underwent single‐bundle ACLR using autologous hamstring grafts and completed a 5‐year follow‐up. Knee function was evaluated preoperatively and at 2 and 5 years postoperatively using the KT‐2000 arthrometer, pivot‐shift test (PST) grade, Lysholm Knee Scoring Scale, International Knee Documentation Committee (IKDC) questionnaire, Knee injury and Osteoarthritis Outcome Score (KOOS), Tegner Activity Scale, and the Anterior Cruciate Ligament‐Return to Sport after Injury (ACL‐RSI) scale. Tibial tunnel diameter was measured via MRI at 1 week, 2 years, and 5 years postoperatively. Graft maturation was evaluated using the signal intensity ratio (SIR). Changes in tunnel diameter and SIR over time were analyzed. Pearson correlation coefficients (*r*) were used to assess the relationship between bone tunnel enlargement (BTE), graft healing, and knee function. Spearman's rank correlation coefficient was used to assess the association between BTE and PST grade.

**Results:**

Tibial tunnel diameter increased from 1 week to 2 years and partially regressed at 5 years, remaining larger than baseline. SIR increased significantly from 1 week to 2 years and decreased slightly by 5 years. At 2 years, tunnel diameter in the tibial tunnel exit (ttE) region was positively correlated with intra‐articular graft SIR (*r* = 0.455, *p* < 0.01), but not with clinical outcomes. By 5 years, no significant correlation was observed between tibial tunnel diameter and graft SIR. However, tibial tunnel diameter in the ttE region was positively correlated with KT‐2000 side‐to‐side difference (SSD) (*r* = 0.411, *p* < 0.05).

**Conclusion:**

Tibial tunnel enlargement progressed until 2 years post‐ACLR, then partially regressed by 5 years. BTE was associated with graft healing at 2 years and with anterior knee stability at 5 years but had no significant adverse impact on long‐term clinical outcomes.

## Graphical abstract

Tibial tunnel diameter increased within 2 years after single‐bundle ACL reconstruction, then slightly decreased by 5 years but remained above baseline. Moderate site‐specific correlations were observed between tunnel enlargement at the tibial tunnel exit, graft maturation at 2 years, and anterior stability at 5 years without long‐term functional impairment.

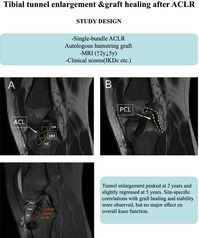

## 1 Introduction

Anterior cruciate ligament reconstruction (ACLR) is an effective surgical treatment for ACL ruptures, involving the fixation of a graft within tibial and femoral bone tunnels. Following surgery, the graft undergoes a healing process involving revascularization and ligamentization within the bone tunnels [1]. The extent of graft healing plays a crucial role in determining postoperative knee function [2]. Meanwhile, bone tunnel enlargement (BTE) is a common complication after ACLR, which may compromise graft positioning, fixation, and biological incorporation, thereby increasing the risk of knee laxity and graft failure [3, 4, 5, 6]. Once graft failure occurs as a result of BTE, revision surgery becomes considerably more challenging. Excessive tunnel enlargement frequently renders one‐stage revision unfeasible, making bone grafting followed by a staged procedure necessary, which further increases surgical complexity and technical demands [7, 8]. In addition, the success rate of revision surgery is markedly reduced [6]. Collectively, these factors not only elevate surgical risks but also impose greater economic costs [9]. Current research suggests that BTE results from the combined effects of biological and biomechanical factors [10]. A mismatch between the graft and the bone tunnel diameter is considered one of the main causes of this phenomenon [11]. Such a mismatch may lead to excessive graft motion and uneven stress distribution within the tunnel, thereby resulting in BTE [10]. The postoperative tendon‐to‐bone healing process and local inflammatory response play a crucial role in BTE development [12]. ACL injury triggers a local nonspecific inflammatory response within the joint, and ACLR further exacerbates this reaction, resulting in markedly elevated concentrations of inflammatory mediators such as interleukin (IL)‐β, IL‐6, and tumor necrosis factor (TNF)‐α in the synovial fluid [13]. The increased levels of these mediators stimulate osteoclast activity, leading to bone resorption and further progression of BTE [14]. When the graft‐to‐tunnel gap is excessive, inflammatory factors can more easily infiltrate the space, further impairing tendon‐to‐bone healing and worsening BTE [15, 16]. Postoperative micromotion, including “bungee” and “windshield wiper” effects, stimulates osteoclast activity and accelerates BTE [17]. Meanwhile, BTE negatively impacts graft tendon‐to‐bone healing [14].

Although previous research has primarily focused on femoral tunnel enlargement given its impact on rotational stability [18, 19, 20], the implications of tibial tunnel enlargement remain underexplored. Indeed, the incidence of tibial tunnel enlargement has been reported to be higher than that of femoral tunnel enlargement [11]. Notably, tibial tunnels tend to experience greater enlargement than femoral tunnels due to gravity‐induced synovial fluid influx and relatively lower tibial bone density [21, 22]. Currently, few studies have specifically explored the relationship between BTE and graft healing, and mid‐ to long‐term follow‐up investigations examining the associations among tibial tunnel morphological changes, graft maturation, and clinical functional outcomes remain scarce.

The purpose of this study was to: (i) retrospectively analyze morphological changes of the tibial tunnel at intraoperative, 1 week, 2 years, and 5 years after single‐bundle ACLR using autologous hamstring tendon; (ii) quantitatively evaluate graft maturation at different tunnel regions using the signal intensity ratio (SIR) on magnetic resonance imaging (MRI) at 1 week, 2 years, and 5 years postoperatively; (iii) investigate the associations between tibial tunnel enlargement, graft healing, and clinical outcomes, including KT‐2000 arthrometer, pivot‐shift test (PST) grade, Lysholm score, International Knee Documentation Committee (IKDC) questionnaire, Knee injury and Osteoarthritis Outcome Score (KOOS), Tegner activity score, and the Anterior Cruciate Ligament‐Return to Sport after Injury (ACL‐RSI) scale. It was hypothesized that smaller tibial BTE would be associated with better graft maturation, offering theoretical implications for surgical and rehabilitative strategies to optimize postoperative outcomes.

## 2 Methods

This study was approved by the Institutional Ethics Committee (Approval No.: 202210). Given the retrospective nature of the study, the requirement for written informed consent from participants was waived. As a retrospective cohort study, the sample size was determined by the eligible cases that met the inclusion criteria during the study period. All included patients underwent primary single‐bundle ACL reconstruction using an autologous hamstring tendon graft with suspensory fixation. A follow‐up was conducted on 43 patients who underwent ACLR at a sports medicine center between January 2015 and January 2017 and met the following inclusion and exclusion criteria.

The inclusion criteria were (1) complete ACL rupture confirmed by arthroscopic examination; (2) no ACL re‐rupture; (3) willingness to participate in the study; (4) intact menisci confirmed intraoperatively, without repair or partial resection. The exclusion criteria were (1) history of previous knee surgery or multi‐ligament injury; (2) postoperative complications such as infection or graft failure; (3) presence of systemic diseases, including rheumatoid arthritis, osteoarthritis, gout, or osteoporosis; (4) history of previous or concurrent injury to the contralateral (nonoperative) knee, to avoid confounding of side‐to‐side difference (SSD) measurements; (5) inability to complete required follow‐up assessments and examinations. Patients were invited for follow‐up assessments via telephone and outpatient visits at designated time points.

### 2.1 Surgical Technique

**FIGURE 1:**
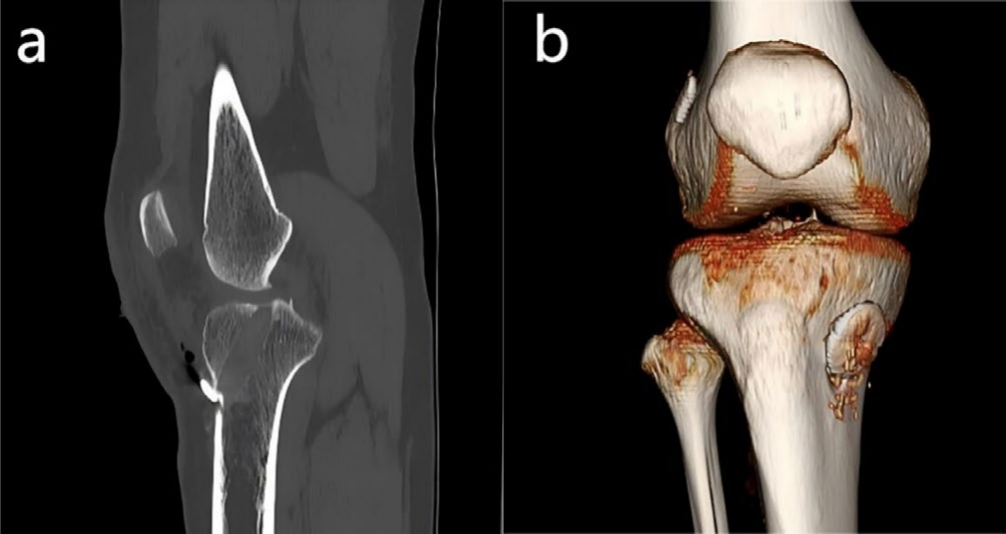
Postoperative computed tomography (CT) images demonstrating tibial tunnel position after single‐bundle anterior cruciate ligament reconstruction. (a) Three‐dimensional volume‐rendered CT reconstruction of the knee showing the tibial tunnel aperture on the anteromedial aspect of the proximal tibia. (b) Sagittal CT reconstruction demonstrating the intraosseous trajectory of the tibial tunnel from the anteromedial cortex to the native ACL footprint on the tibial plateau.

All patients underwent single‐bundle ACLR using autologous hamstring tendon grafts, with surgeries performed by the same senior surgeon. The procedure was conducted with the patient in the supine position, both feet secured, and the knee flexed to 90°. Hamstring tendons were harvested and prepared into an 8‐strand autograft with a diameter of 7–9 mm. The knee was then flexed to 150° to facilitate femoral tunnel placement, and the lateral wall of the intercondylar notch was identified using a medial accessory portal under arthroscopic guidance. Intraoperative fluoroscopy was used to confirm tunnel positioning, with the tibial tunnel centered at the remnant tibial attachment site. For graft fixation, a cortical suspension device was used on the femoral side, while an adjustable‐loop suspension plate was applied on the tibial side. After femoral fixation, the graft was pretensioned using an instrumented tensioning device (Arthrex, Naples, FL, USA). The tibial end of the graft was gradually tensioned to 20 N, and tibial fixation was then performed while maintaining this load. The same tensioning protocol was applied to all patients. To promote graft‐to‐bone healing, all tibial tunnels were filled with autologous bone debris using a standardized technique. No bone packing was performed in the femoral tunnels to maintain consistency in surgical procedures across all patients. To reduce confounding factors, only suspensory fixation was used in all cases, thereby avoiding potential interference from other fixation techniques on tunnel diameter. Postoperative CT scans of the knee were obtained immediately after surgery to confirm tunnel placement. The representative images are shown in Figure 1.

### 2.2 Postoperative Rehabilitation

A protective hinged brace was used for 6 weeks after the operation. All patients were followed according to the standardized rehabilitation protocol of the department. The early range of motion (ROM) was restricted, with passive ROM limited to 90° within the first 2 weeks postoperatively. By the fourth postoperative week, patients were allowed to perform light weight‐bearing activities with crutch assistance. At 6 weeks, the hinged brace was replaced with a soft brace, and patients were encouraged to engage in low‐to‐moderate intensity aerobic activities, such as jogging or swimming, within the first 6 months. Full return to contact sports was recommended between 9 and 12 months postoperatively.

### 2.3 Clinical Assessment

This retrospective study with a 5‐year follow‐up evaluated clinical function preoperatively and at 2 and 5 years postoperatively. The assessment tools included the KT‐2000 arthrometer, Lysholm Knee Scoring Scale, IKDC questionnaire, KOOS, Tegner Activity Scale, and ACL‐RSI scale. All instruments have been validated in the Chinese population [23, 24, 25, 26]. KT‐2000 testing was performed according to a standardized protocol, applying an anterior tibial load of 134 N with the patient in the supine position and the knee flexed at 20°. Each measurement was repeated three times, and the mean value was recorded. The SSD in anterior tibial translation between the operated and contralateral knees was calculated. All clinical assessments were conducted by the same experienced orthopedic surgeon to ensure measurement reliability. In addition, rotational knee stability was assessed using the PST at the 2‐ and 5‐year follow‐up visits. PST was graded on a 4‐point scale (Grade 0–III), where Grade 0 indicated a negative test and Grades I–III indicated increasing degrees of subluxation.

### 2.4 Bone Tunnel Diameter Measurement

**FIGURE 2:**
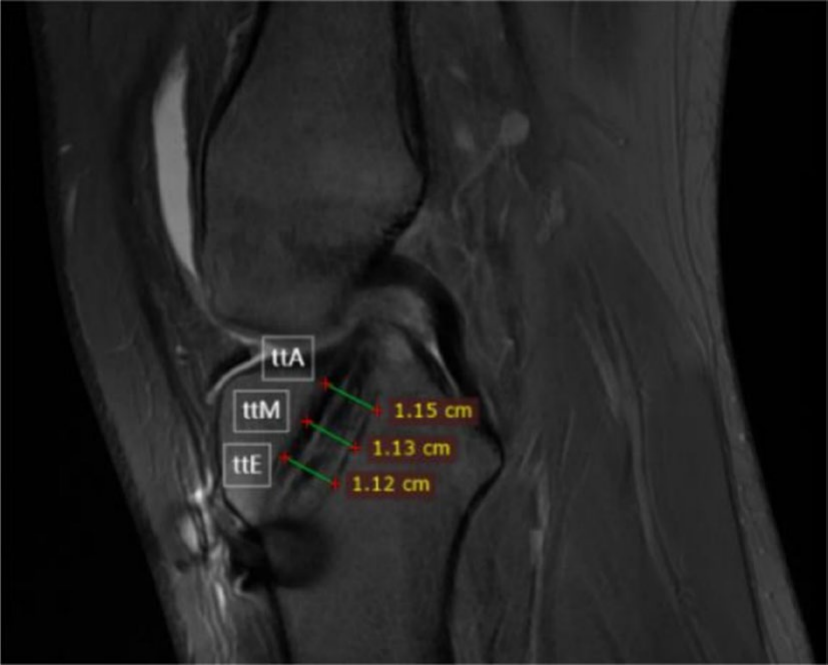
Representative images of the knee demonstrating the locations of tibial tunnel diameter measurements. ttA, tibial tunnel aperture; ttE, tibial tunnel exit; ttM, tibial tunnel midsection.

During follow‐up, the affected knee underwent 3.0 T MRI scanning (GE Signa HDx, USA). Throughout the examination, the knee joint remained in a fixed position, utilizing a fast spin‐echo T2‐weighted sequence (TR/TE: 3000/98 ms; slice thickness/gap: 4.0/1.0 mm; field of view: 16 cm; NEX: 2; ETL: 14; bandwidth: ±31.25 kHz/field of view). The knee was positioned at 10° flexion and externally rotated by 10°–15°. By identifying the plane passing through the center of the tibial tunnel, oblique sagittal images were reconstructed. The clearest image of the tibial tunnel was selected for analysis. The tibial tunnel was divided into three sections based on its anatomical location: the tibial tunnel aperture (ttA), the tibial tunnel midsection (ttM), and the tibial tunnel exit (ttE). The measurement points were defined as follows: ttA was measured 0.5 cm from the tunnel entrance, ttM was measured at the midpoint along the tunnel's longitudinal axis, and ttE was measured 0.5 cm from the tunnel exit [3]. The widths of these three sections were measured using RadiAnt DICOM Viewer software (version 2023.1, Poland) (Figure 2). MRI examinations were performed at 1 week, 2 years, and 5 years postoperatively to assess the tibial tunnel diameter. The initial tunnel diameter at the time of drilling was determined retrospectively from surgical records, where the diameters of ttA, ttM, and ttE were identical. All tibial tunnel diameter measurements were performed by two experienced orthopedic surgeons using a standardized protocol. The observers were not blinded to the patients' clinical information. Each observer performed the tunnel width measurements on two separate occasions, and for each occasion three repeated measurements were obtained at each section and averaged to yield that observer's value. For statistical analysis, the mean values of the two observers' measurements were used. Intra‐ and interobserver reliability were assessed using intraclass correlation coefficients (ICCs) and the standard error of measurement (SEM).

### 2.5 Graft Healing Assessment

On oblique sagittal MRI images, three 0.5 cm^2^ regions of interest (ROIs) were selected along the intra‐articular portion of the graft at the ttA, ttM, and ttE sections. The signal intensity (SI) of each ROI was measured. Additionally, an ROI was placed in the posterior cruciate ligament (PCL) to measure its signal intensity. Graft healing was assessed by calculating the SIR, defined as the SI of the ACL graft at each section divided by the SI of the PCL. The PCL was selected as the reference structure because it is an intra‐articular ligament with relatively stable morphology and orientation and is not directly affected by ACL reconstruction. It lies adjacent to the graft on the same sagittal slices, so both structures are imaged under identical scanning conditions, and no PCL abnormalities were observed in this cohort. Using the PCL as an internal reference on the same slice helps to minimize the influence of scanner settings and interscan variability on signal intensity measurements. MRI scans were performed at 1 week, 2 years, and 5 years postoperatively. All imaging data were independently reviewed by two senior orthopedic surgeons.

**FIGURE 3:**
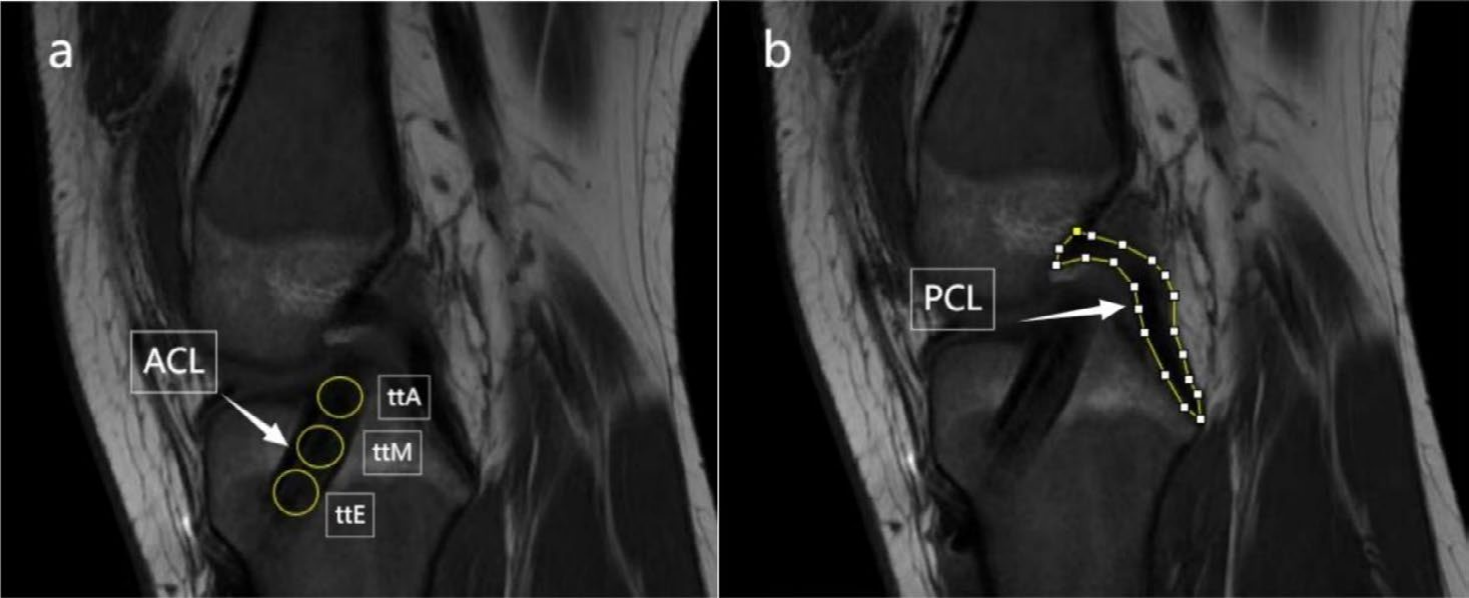
Selecting ROIs on MRI to measure the signal intensity of the ACL graft (a) and the PCL (b). ACL, anterior cruciate ligament; ttA, tibial tunnel aperture; ttE, tibial tunnel exit; ttM, tibial tunnel midsection; PCL, posterior cruciate ligament; ROI, region of interest.

On T1‐weighted images, the ACL graft at ttA, ttM, and ttE, as well as the PCL, was outlined using ImageJ software (version 1.51j8) (Figure 3). These outlined areas were designated as ROIs, and the mean SI of each ROI was recorded. To account for intra‐articular pathological conditions such as inflammation or effusion, standardized SI measurements were used to eliminate confounding effects. The SIR was calculated as follows: *SIR = ACL graft signal intensity/PCL signal intensity* [27, 28]. Higher SIR values indicate poorer graft healing. All graft SIR measurements were performed by two senior orthopedic surgeons using the same standardized protocol described above. The observers were not blinded to the patients' clinical information. Each observer performed ROI selection and signal intensity measurements on two separate occasions at least 2 weeks apart, and for each occasion three repeated measurements were obtained at each section and averaged to yield that observer's value. For statistical analysis, the mean of the two observers' measurements was used. Intra‐ and interobserver reliability for SIR were assessed using ICCs and SEM.

### 2.6 Statistical Analysis

Descriptive statistical analysis was performed for patient characteristics. The normality of continuous variables was assessed using the Shapiro–Wilk test. Data were expressed as the mean ± SD. Repeated‐measures analysis of variance (ANOVA) was used to compare the diameter and graft SIR of each part of the tibial tunnel at intraoperative, 1‐week, 2‐year, and 5‐year time points. Correlations between tibial tunnel diameter, clinical outcomes, and graft SIR were analyzed using Pearson product moment correlation coefficients (*r*). Associations between PST grade (ordinal, 0–II) and tibial tunnel diameters at ttA, ttM, and ttE were assessed using Spearman's rank correlation at 2 and 5 years postoperatively. A *p*‐value < 0.05 was considered statistically significant. SPSS software (Version 25.0; IBM Corp., Armonk, NY, USA) was used for all statistical analyses.

## 3 Results

Of the 43 eligible patients, 8 were excluded due to postoperative complications or loss to follow‐up, leaving 35 patients who completed the full 5‐year follow‐up and were included in the final analysis. (Table 1). Mean age was 35.2 ± 8.2 years, average height 174.1 ± 7.2 cm, weight 75 ± 11.9 kg, and BMI 24.7 ± 3.3 kg/m^2^. Most surgeries involved the left knee (65.7%). Patients included 12 females (34.3%) and 23 males (65.7%).

**TABLE 1:** The basic characteristics of the participants.

Patient demographics	Mean ± SD or *n* (%)
No. of patients	35
Age, y	35.2 ± 8.2
Height, cm	174.1 ± 7.2
Weight, kg	75 ± 11.8
BMI, (kg/m^2^)	24.7 ± 3.3
Side, *n*	
Left	23 (65.7)
Right	12 (34.3)
Sex, *n*	
Female	12 (34.3)
Male	23 (65.7)

*Note:* Values are shown as mean ± standard deviation unless otherwise indicated.

Abbreviation: BMI, body mass index.

### 3.1 Clinical Outcomes

A statistical analysis of follow‐up clinical outcomes was performed (Table 2). Clinical functional scores improved significantly over time. KT‐2000 values decreased significantly at 2 years, slightly increasing at 5 years. At the 2‐year follow‐up, KT‐2000 side‐to‐side differences were small (0.46 ± 0.85 mm; range −2 to 3 mm), and PST showed Grade 0 in 29 patients and Grade I in 6 patients. At the 5‐year follow‐up, 23 patients had Grade 0, 9 patients had Grade I, and 3 patients had a Grade II PST; no grade III PST was observed.

**TABLE 2:** Clinical functional outcomes of participants.

Clinical functional outcomes	Preoperative	2 years	5 years
IKDC score	44.97 ± 5.06	84.14 ± 10.75	96.07 ± 4.22
Lysholm score	50.06 ± 6.18	83.29 ± 15.32	95.69 ± 2.58
Tegner score	4.94 ± 1.03	5.89 ± 1.45	7.66 ± 0.97
KOOS	38.66 ± 7.84	84.11 ± 10.21	94.91 ± 2.88
ACL‐RSI scale	46.82 ± 12.41	68.36 ± 18.38	88.26 ± 7.97
KT2000 SSD	4.09 ± 1.42	0.46 ± 0.85	0.49 ± 0.78
PST positive (GRADE ≥ I), *n* (%)	35 (100)	6 (17.1)	12 (34.2)

*Note:* Data are presented as mean ± standard deviation unless otherwise indicated. PST values are shown as *n* (%). PST positive was defined as grade ≥ I on the four‐point PST scale (Grade 0–III).

Abbreviations: ACL‐RSI Scale, Anterior Cruciate Ligament‐Return to Sport after Injury Scale; IKDC, International Knee Documentation Committee; KOOS, Knee injury and Osteoarthritis Outcome Score; PST, Pivot‐shift Test; SSD, Side‐to‐Side Difference.

### 3.2 Tibial Tunnel Diameter Measurements

**FIGURE 4:**
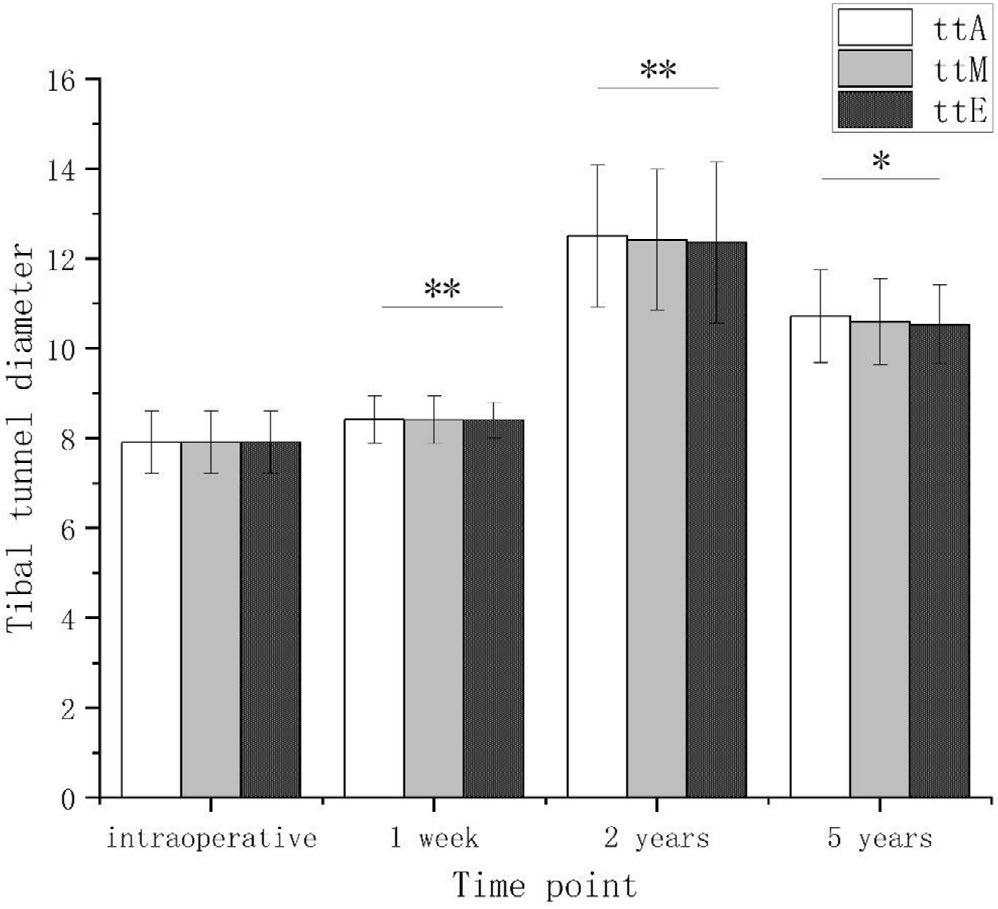
The tibial tunnel diameter at the aperture (ttA), midsection (ttM), and exit (ttE) was measured intraoperatively, and at 1 week, 2 years, and 5 years postoperatively. *p < 0.05, **p < 0.01 compared with the previous time point. At the same time point, no significant differences in diameter were observed among the three tunnel sections (*p* > 0.05).

Tibial tunnel diameters (ttA, ttM, and ttE) were measured intraoperatively, and at 1 week, 2, and 5 years postoperatively. In the ttA region, the initial intraoperative diameter was 7.91 ± 0.69 mm, which slightly increased to 8.41 ± 0.53 mm at 1 week postoperatively. By the second postoperative year, the diameter had significantly enlarged to 12.51 ± 1.58 mm before slightly decreasing to 10.72 ± 1.03 mm at the fifth year. A similar pattern was observed in the ttM region, where the initial diameter was 7.91 ± 0.69 mm, increasing to 8.41 ± 0.53 mm at 1 week, peaking at 12.42 ± 1.57 mm at 2 years, and then decreasing to 10.60 ± 0.96 mm at 5 years. The ttE region exhibited a comparable trend, with an initial diameter of 7.91 ± 0.69 mm, increasing to 8.40 ± 0.39 mm at 1 week, reaching a peak of 12.36 ± 1.79 mm at 2 years, and slightly decreasing to 10.53 ± 0.88 mm at 5 years (Figure 4). The intra‐observer ICC for tunnel diameter measurement was 0.92, with a SEM of 0.18 mm, and the inter‐observer ICC was 0.89, indicating excellent measurement reliability. Therefore, the mean values of the two observers' measurements were used for analysis. Although the mean diameter at the ttA region increased by approximately 4.6 mm between the intraoperative measurement and the 2‐year follow‐up, this enlargement was not associated with overt ACL graft insufficiency: patients with graft failure or ACL re‐rupture were excluded from the cohort, KT‐2000 side‐to‐side differences remained within the clinically acceptable range, and no high‐grade (Grade ≥ II) PST was observed at this time point.

### 3.3 Graft SIR Measurements

**FIGURE 5:**
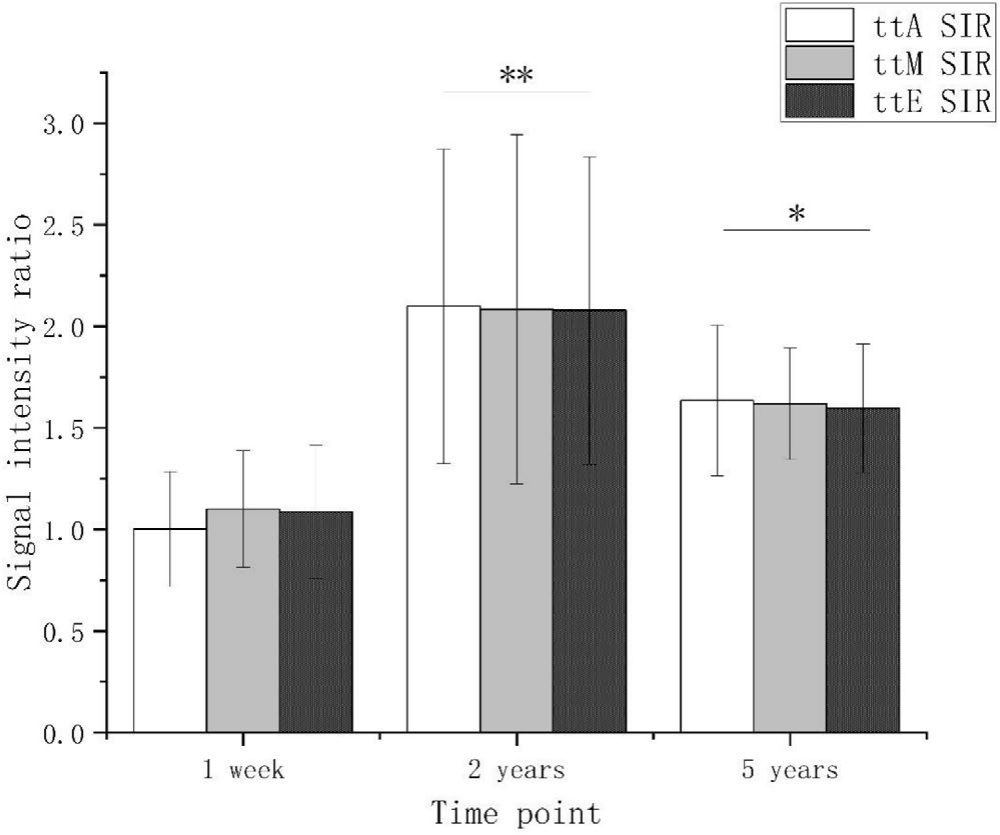
The mean signal intensity ratio (SIR) of the tibial tunnel aperture (ttA), midsection (ttM), and exit (ttE) was measured at 1 week, 2 years, and 5 years postoperatively. *p < 0.05, **p < 0.01 compared with the previous time point. At the same time point, no significant differences in graft SIR were found among the three tunnel sections (*p* > 0.05).

Graft SIR varied significantly over time across all tunnel regions. In the ttA region, the SIR increased significantly from 1.00 ± 0.29 at 1 week to 2.10 ± 0.77 at 2 years, followed by a decrease to 1.64 ± 0.37 at 5 years. A similar trend was observed in the ttM region, where the SIR increased from 1.10 ± 0.29 at 1 week to 2.08 ± 0.86 at 2 years, then declined to 1.61 ± 0.28 at 5 years. In the ttE region, the SIR rose from 1.09 ± 0.33 at 1 week to 2.08 ± 0.76 at two years before decreasing to 1.60 ± 0.32 at 5 years (Figure 5). The intra‐observer ICC for SIR measurement was 0.90, with a SEM of 0.05, and the inter‐observer ICC was 0.87, also indicating high reliability. Accordingly, the mean values of the two observers' measurements were used for subsequent analyses.

### 3.4 Correlation Analysis of Tibial Tunnel Enlargement and Clinical Outcomes

At the 2‐year follow‐up, no significant correlation was found between tibial tunnel diameter and clinical outcomes (*p* > 0.05) (Table 3). However, at the 5‐year follow‐up, a moderate, statistically significant positive correlation was observed between ttE tibial tunnel diameter and KT‐2000 SSD (*r* = 0.411, *p* = 0.014) (Table 4). In addition, Spearman rank analysis demonstrated no significant associations between PST grade and tibial tunnel enlargement at any tunnel region at either 2 or 5 years (*p* > 0.05) (Table 5).

**TABLE 3:** Correlation analysis of tibial tunnel diameters versus clinical outcomes at 2 years.

	IKDC score		Lysholm score		Tegner score		KOOS		ACL‐RSI scale		KT2000 SSD	
	*r*	*p*	*r*	*p*	*r*	*p*	*r*	*p*	*r*	*p*	*r*	*p*
ttA	−0.180	0.302	−0.043	0.805	0.002	0.993	0.103	0.555	0.188	0.279	0.045	0.797
ttM	0.029	0.868	0.113	0.517	−0.022	0.900	0.172	0.322	0.157	0.367	0.194	0.263
ttE	−0.080	0.647	0.002	0.992	−0.014	0.938	0.106	0.543	0.075	0.669	0.100	0.566

Abbreviations: ACL‐RSI Scale, Anterior Cruciate Ligament‐Return to Sport after Injury Scale; IKDC, International Knee Documentation Committee; KOOS, Knee injury and Osteoarthritis Outcome Score; SSD, Side‐to‐Side Difference; ttA, tibial tunnel aperture; ttE, tibial tunnel exit; ttM, tibial tunnel midsection.

**TABLE 4:** Correlation analysis of tibial tunnel diameters versus clinical outcomes at 5 years.

	IKDC score		Lysholm score		Tegner score		KOOS		ACL‐RSI scale		KT2000 SSD	
	*r*	*p*	*r*	*p*	*r*	*p*	*r*	*p*	*r*	*p*	*r*	*p*
ttA	0.180	0.301	−0.226	0.191	0.196	0.260	−0.230	0.185	0.174	0.319	0.297	0.083
ttM	0.160	0.359	−0.146	0.402	0.206	0.236	−0.221	0.201	0.281	0.102	0.282	0.101
ttE	0.216	0.213	−0.208	0.231	0.182	0.294	−0.333	0.051	0.202	0.243	0.411	**0.014** *

*Note:* Bold values indicate statistically significant differences (*p* < 0.05).

Abbreviations: ACL‐RSI Scale, Anterior Cruciate Ligament‐Return to Sport after Injury Scale; IKDC, International Knee Documentation Committee; KOOS, Knee injury and Osteoarthritis Outcome Score; SSD, Side‐to‐Side Difference; ttA, tibial tunnel aperture; ttE, tibial tunnel exit; ttM, tibial tunnel midsection.

* *p* < 0.05.

**TABLE 5:** Spearman correlation analysis of tibial tunnel diameters versus PST grade at 2 and 5 years.

	2 years		5 years	
	*ρ*	*p*	*ρ*	*p*
ttA	−0.011	0.949	0.148	0.395
ttM	−0.004	0.983	−0.019	0.915
ttE	−0.165	0.343	0.003	0.988

Abbreviations: PST, Pivot‐shift test; ttA, tibial tunnel aperture; ttE, tibial tunnel exit; ttM, tibial tunnel midsection.

### 3.5 Correlation Analysis of Tibial Tunnel Enlargement and Graft SIR

Additionally, at the 2‐year follow‐up, tunnel diameters at the ttA and ttM regions were not significantly correlated with the intra‐articular graft SIR (*p* > 0.05). However, the ttE region diameter showed a moderate, statistically significant positive correlation with the intra‐articular graft SIR (*r* = 0.455, *p* = 0.006) (Table 6). At the 5‐year follow‐up, no significant correlation was observed between graft SIR and tunnel diameters (*p* > 0.05) (Table 7).

**TABLE 6:** Correlation analysis of tibial tunnel diameters versus Graft SIR at 2 years.

	ttA SIR		ttM SIR		ttE SIR	
	*r*	*p*	*r*	*p*	*r*	*p*
ttA	0.186	0.285				
ttM			0.274	0.112		
ttE					0.455	**0.006** **

*Note:* Bold values indicate statistically significant differences (*p* < 0.05).

Abbreviations: SIR, signal intensity ratio; ttA, tibial tunnel aperture; ttE, tibial tunnel exit; ttM, tibial tunnel midsection.

** *p* < 0.01.

**TABLE 7:** Correlation analysis of tibial tunnel diameters versus graft SIR at 5 years.

	ttA SIR		ttM SIR		ttE SIR	
	*r*	*p*	*r*	*p*	*r*	*p*
ttA	0.100	0.567				
ttM			0.180	0.301		
ttE					0.138	0.429

Abbreviations: SIR, signal intensity ratio; ttA, tibial tunnel aperture; ttE, tibial tunnel exit; ttM, tibial tunnel midsection.

## 4 Discussion

This study showed that tibial tunnel enlargement increased up to 2 years post‐ACLR, then decreased by 5 years, although it remained above baseline. The correlation between early BTE and graft maturation weakened over time, whereas an association with knee stability was observed at the later follow‐up stage. These findings suggest a shift in the clinical implications of BTE over time.

### 4.1 Temporal and Regional Characteristics of Tibial Tunnel Enlargement

In the present study, tibial tunnel diameter increased up to 2 years after ACLR and then gradually decreased by 5 years, although it remained above the intraoperative value. Our findings suggest that BTE follows a pattern of rapid enlargement in the early postoperative period, followed by gradual slowing and stabilization. This temporal pattern is generally consistent with previous studies, which reported that BTE typically peaks at 3–6 months, stabilizes by 2 years, and remains enlarged thereafter [3, 23]. Optimal tunnel diameter is essential for graft healing and function [2]. Excessive narrowing can disrupt stress distribution, which hinders graft healing and negatively impacts clinical outcomes [29]. Tunnel remodeling is constrained by the size of the implanted graft and the original drilling diameter [30, 31]. While tunnel enlargement peaks in the early postoperative period and gradually decreases over time, the diameter fails to return to the size of the originally drilled tunnel. After ACL reconstruction, an initial phase of osteoclastic bone resorption driven by graft micromotion, stress shielding, and exposure to synovial fluid and inflammatory mediators promotes tunnel expansion [11]. As the graft matures and the mechanical environment stabilizes, a reparative phase with new bone formation and cortical thickening may lead to gradual narrowing of the tunnel lumen [32]. However, early cancellous bone loss, persistent stress‐shielded areas, and fibrous tissue within the tunnel limit complete re‐ossification, so the tunnel diameter decreases but does not return to its original size [33]. Although this interpretation is in line with current concepts of bone remodeling, the mechanisms underlying this incomplete regression are not yet fully elucidated and warrant further investigation [23].

Regional differences provide additional insights into BTE. Although the tibial tunnel initially appears uniform, regional differences in enlargement emerge over time [34]. Biomechanically, uneven stress distribution within the tunnel results in differing contact pressures between the graft and the tunnel wall, with the ttA region experiencing the highest load and therefore being the most susceptible to BTE [35]. A prospective study by Weber et al. [3] reported that when absorbable screws were used for tibial fixation, BTE and graft abrasion were more frequently observed at ttA, while ttE exhibited minimal BTE. Similarly, Ohori et al. [34] reported greater BTE at ttA with suspensory fixation, further supporting the idea that mechanical stress plays a critical role. The results of this study demonstrated a similar trend, although it did not reach statistical significance [11]. Biologically, synovial fluid may accumulate more easily in the distal part of the tibial tunnel under the effect of gravity, increasing local exposure to inflammatory mediators and other catabolic factors. This mechanism may be particularly relevant at the ttE region, where fluid retention is more likely [21]. In contrast, biomechanical influences appear to follow the opposite pattern, with greater graft–tunnel wall contact stress and micromotion toward the proximal tunnel region [35]. Therefore, the regional pattern of BTE is likely shaped by both mechanisms rather than by a single factor alone. In our cohort, the observed spatial distribution of BTE may reflect the combined effects of mechanically driven stress and biologically mediated tunnel remodeling acting in opposite directions [11].

### 4.2 Association Between Tibial Tunnel Enlargement and Graft Maturation

In our cohort, a moderate, site‐specific association between BTE and graft maturation was observed at the ttE region at 2 years, whereas this association weakened over time. Full graft ligamentization may exceed a decade, with critical changes within 2 years [36]. This timeframe coincides with the period of BTE progression. During this early phase, reduced micromotion at the graft‐tunnel interface helps limit inflammatory exposure, thereby promoting graft healing and reducing BTE progression [37, 38]. This 2‐year ttE‐specific association is consistent with the findings of Putnis et al. [39], who reported that lower SIR was associated with reduced BTE. The PCL served as a relatively stable internal reference ligament with no detectable abnormalities on serial MRIs, which helped reduce the impact of scanner‐related variability on SIR measurements. These findings suggest that limiting excessive BTE may be beneficial for graft healing, particularly during the early postoperative remodeling phase. Suspensory fixation leads to less BTE and better healing at ttE, under lower mechanical stress. In contrast, ttA and ttM sites, subject to greater load and micromotion, showed weaker or absent correlations due to interference from rehabilitation and activity levels [40, 41, 42]. This further supports the stronger association observed at the ttE site. By 5 years, graft ligamentization nearly completes, resembling native ACL on MRI [43]. Graft healing and tunnel remodeling stabilize, and BTE‐graft correlation weakens in the long term.

### 4.3 Association Between Tibial Tunnel Enlargement and Clinical Outcomes

BTE showed no significant association with most functional outcome scores at 5 years, whereas a moderate positive correlation was observed between ttE tunnel enlargement and KT‐2000 measurements. These findings suggest a limited overall functional impact of BTE in this cohort, with a site‐specific association with anterior knee laxity. Previous studies have suggested that excessive tibial tunnel enlargement may negatively affect graft healing and joint stability, potentially increasing the risk of graft failure upon return to sport [44, 45]. Most studies show limited short‐term clinical impact, though some find associations with functional performance [46]. Short‐term rehabilitation may mask early negative effects of BTE [10, 47, 48], explaining the limited impact. In our cohort, although the ttA diameter increased by more than 4 mm at 2 years compared with the intraoperative measurement, KT‐2000 side‐to‐side differences remained low in the vast majority of patients and no high‐grade PST was detected, indicating that this degree of tunnel enlargement was not necessarily accompanied by clinically evident ACL graft insufficiency. Spearman's rank correlation analysis also demonstrated no significant associations between PST grade and tibial tunnel enlargement at any region at either 2 or 5 years, suggesting that, within the magnitude of BTE observed in this cohort, the effect on rotational knee stability was limited. The KT‐2000 measurements associated with ttE tunnel enlargement remained within the clinically normal ranges (−1 to 2 mm) [49]. Thus, BTE at ttE may minimally affect anterior–posterior stability, but does not impair knee function. Anatomical factors make ttE more sensitive to shear forces [35, 47, 50]. KT‐2000's sensitivity to shear may explain this result [51].

### 4.4 Limitations and Prospect

This study has several limitations. First, it was a single‐center retrospective study with a relatively small sample size (*n* = 35), which may introduce selection bias and limit the generalizability of the findings. As all eligible patients during the study period were included and no formal a priori power calculation was performed, the present analysis should be regarded as exploratory, with limited power to detect small effects or support multivariable models. Second, the mid‐term follow‐up restricted the assessment of postoperative changes across different stages, and some interpretations relied on previous literature. Third, the imaging protocol and analysis were specifically focused on tibial tunnel morphology, and femoral tunnel enlargement was not quantified using standardized imaging; consequently, femoral tunnel changes could not be systematically evaluated in this cohort. In addition, all imaging measurements were performed by two nonblinded observers, which may have introduced measurement bias despite the good intra‐ and interobserver reliability indicated by the ICCs and the use of mean values from both observers. Finally, correlations between KT‐2000 measurements or graft maturation and tunnel enlargement were generally weak to moderate and were not adjusted for potential confounding variables, so these associations should be interpreted with caution and require confirmation in larger, long‐term studies.

Despite these limitations, the present study has notable strengths, particularly the 5‐year follow‐up. Although BTE severity was associated with knee laxity at 5 years, its overall impact on clinical function was limited. However, it should be noted that this finding is specific to the cohort. Longer follow‐up may reveal continued laxity progression and sustained BTE association [52]. Future studies require larger samples, multicenter designs, and longer follow‐ups to clarify BTE impacts.

From the perspective of applicability and future research, all patients underwent tibial suspensory fixation. Compared to screw fixation, suspensory systems reduce stress transmission and provide early load buffering [34, 40], lowering BTE incidence [53, 54]. Nonetheless, mechanical limitations persist, including the “bungee” and “windshield wiper” effects, both of which may lead to excessive graft micromotion within the tunnel [11, 55]. Despite widespread use, long‐term data on suspensory fixation remain limited, highlighting the need for further comparative studies.

## 5 Conclusion

Tibial tunnel diameter increased gradually within 2 years following single‐bundle ACL reconstruction using autologous hamstring tendon, with a slight reduction observed by 5 years. Moderate and site‐specific correlations were found between tunnel enlargement at the ttE region and graft signal intensity at 2 years, as well as anteroposterior knee stability at 5 years. However, these correlations were isolated and did not consistently affect overall clinical outcomes. Thus, these findings suggest potential localized relationships between tibial tunnel morphology and postoperative recovery, but further research is needed to determine the long‐term clinical significance of bone tunnel enlargement.

## Author Contributions

Conceptualization: Wenbo Tang, Jingbin Zhou; Methodology: Tao Li, Xiaohan Zhang; Formal analysis and investigation: Wenbo Tang, Bingying Zhang, Feng Qu, Mingze Liu; Writing – original draft preparation: Wenbo Tang, Xiaohan Zhang, Bingying Zhang, Jingyi Sun; Writing – review and editing: Wenbo Tang, Jingbin Zhou, Tao Li, Xiaohan Zhang, Bingying Zhang, Feng Qu, Mingze Liu, Feng Gao; Supervision: Jingbin Zhou, Feng Gao.

## Funding

The authors have nothing to report.

## Disclosure

All authors listed meet the authorship criteria according to the latest guidelines of the International Committee of Medical Journal Editors (ICMJE), and all authors are in agreement with the manuscript.

## Ethics Statement

This study was approved by the Ethics Committee of the National Institute of Sports Medicine, General Administration of Sport of China (approval number: 202210).

## Conflicts of Interest

The authors declare no conflicts of interest.

## Data Availability

The data that support the findings of this study are available from the corresponding author upon reasonable request.
